# A Case of Macrolide-Refractory* Mycoplasma pneumoniae* Pneumonia in Pregnancy Treated with Garenoxacin

**DOI:** 10.1155/2017/3520192

**Published:** 2017-12-10

**Authors:** Yoko Matsuda, Yoshitsugu Chigusa, Eiji Kondoh, Isao Ito, Yusuke Ueda, Masaki Mandai

**Affiliations:** ^1^Department of Gynecology and Obstetrics, Kyoto University, 54 Shogoin Kawahara-cho, Sakyo-ku, Kyoto 606-8507, Japan; ^2^Department of Respiratory Medicine, Kyoto University, 54 Shogoin Kawahara-cho, Sakyo-ku, Kyoto 606-8507, Japan

## Abstract

Pneumonia in pregnancy is associated with adverse maternal and foetal outcomes, and intensive treatment with appropriate antibiotics is essential. However, cases caused by pathogens that are resistant to antibiotics suitable for the developing foetus are challenging. We herein report a case of macrolide-refractory* Mycoplasma pneumoniae* pneumonia in pregnancy. A 40-year-old multigravida with twin pregnancy complained of cough and fever at 13 weeks of gestation and was diagnosed with pneumonia. Even though empiric treatment with ceftriaxone and oral azithromycin was started, her condition deteriorated rapidly. The findings of chest computed tomography suggested* Mycoplasma pneumoniae* pneumonia. Since azithromycin did not work, this strain was considered to be macrolide-refractory. Garenoxacin, an oral quinolone, was selected and was dramatically effective. The use of quinolone could be justified with the emergence of drug-resistant bacterial/atypical pneumonia and in the maternal life-threatening condition.

## 1. Introduction

Pneumonia in pregnant women can become severe and is the most common nonobstetric infection contributing to maternal mortality in the peripartum period. In the recent years, concern has arisen regarding pathogens of pneumonia which are resistant to the initial empiric treatment such as*β*-lactam antibiotics. The treatment strategy of drug-resistant pneumonia is challenging, especially in pregnant patients, because several kinds of promising antibiotics are considered to be avoided in pregnancy. Here, we describe a case of macrolide-refractory* Mycoplasma pneumoniae* pneumonia at 13 weeks of gestation. The administration of Garenoxacin, an oral quinolone antibiotic, dramatically ameliorated severe cough, dyspnoea, and fever, and the patient recovered quickly. To the best of our knowledge, this is the first case in which Garenoxacin was administered to a pregnant woman. We also discuss the rationale and safety of quinolone usage in pregnancy based on the relevant literatures.

## 2. Case Report

**Figure 1:**
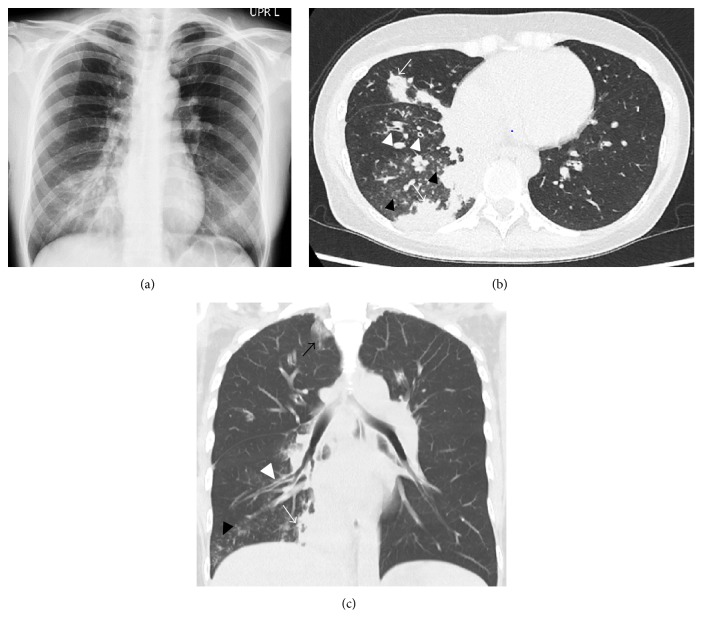
Chest radiography (a), axial view (b), and coronal view (c) of chest computed tomography (CT) images of the case. The chest X-ray showed a high-density area in right lower lobe. The chest CT revealed airspace consolidations (white arrows), thickening of bronchial walls (white triangles), and numerous centrilobular nodules (black triangles). The coronal view showed ground-glass opacity in the right upper lobe (black arrow).

A 40-year-old female, gravida 3, para 1, with an unremarkable past medical history conceived with in vitro fertilization. At 12 weeks and 5 days of gestation, she was referred to our hospital with the diagnosis of dichorionic diamniotic twin pregnancy. She complained of slight general fatigue and anorexia. Two days later, at 13 weeks and 0 days of gestation, she visited us again with a complaint of unproductive cough and fever. Regarding the obstetrical examination, she had no abnormal findings. Her vaginal secretions were yellowish, and her cervix was closed. Transvaginal ultrasound showed 5 cm of cervical length and normal heart beats of two foetuses. Her vital signs were as follows: body temperature, 38.1°C, pulse, 102 beats per minute, blood pressure, 101/67 mmHg, SpO_2_, 93% (room air), and respiratory rate, 25 per minute. The laboratory data are shown in Table 1. Although she had persistent cough, her chest auscultation findings were subtle; only weak inspiratory wheeze was appreciated. Even though* Streptococcus pneumoniae* urinary antigen test was negative, the chest X-ray revealed infiltrate with bronchial tram lines in right lower lobe (Figure 1(a)). Therefore, pneumonia was suspected and empiric therapy was commenced with 1 g of ceftriaxone every 12 hours and single administration of 2 g of oral azithromycin in order to cover both bacterial and nonbacterial pathogens of community-acquired pneumonia. Nasal oxygenation was also employed to keep her SpO_2_ above 95%. Despite the antibiotic therapy for 72 hours, her fever was not alleviated, and she remained with cough and dyspnoea and deteriorated rapidly. The chest computed tomography (CT) revealed airspace consolidation, ground-glass opacity, centrilobular nodules, and thickening of the bronchial walls (Figures 1(b) and 1(c)), which are distinctive features of the CT findings of* Mycoplasma pneumoniae* pneumonia [1]. Combined with the fact that azithromycin did not work, macrolide-resistant* Mycoplasma pneumoniae* pneumonia was suspected. Thus, after consultation with a chest physician, oral administration of 400 mg of Garenoxacin every 24 hours was started at 13 weeks and 3 days of gestation. Shortly after Garenoxacin administration, dyspnoea dramatically ameliorated, and fever began to subside within 24 hours. She took Garenoxacin for seven days and was discharged from the hospital at 14 weeks and 6 days of gestation. Eventually, she was diagnosed with* Mycoplasma pneumoniae* pneumonia based on the serological test;* Mycoplasma* particle agglutination titres increased from 1 : 80 to 1 : 2,560 (32 times) in seven days. The subsequent pregnancy course was uneventful; because the first foetus presented as breech, she delivered twins by caesarean section at 37 weeks and 5 days of gestation. The twin male babies weighed 2402 g and 2404 g, respectively, and had no abnormal findings. There were no signs of infection, either.

## 3. Discussion

Pneumonia is a relatively uncommon complication of pregnancy arising in 0.78 to 2.7 per 1,000 deliveries. The most common aetiological agents of pneumonia in pregnancy are typical bacterial pathogens, such as* Streptococcus pneumoniae* and* Haemophilus influenzae.* Additionally, atypical bacterial pathogens, including* Mycoplasma pneumoniae*,* Chlamydia pneumoniae*, and* Legionella pneumophilia*, are also identified. Although antibiotic therapy has advanced, pneumonia in pregnancy can be associated with considerable maternal mortality and morbidity [2]. A change in maternal cellular immunity, namely, immunosuppression caused by pregnancy or maternal physiologic changes such as a decrease in the functional residual capacity of lungs, may be related to an unfavourable course of pneumonia in pregnant women. Furthermore, pneumonia during gestation is associated with adverse pregnancy outcomes. Preterm labour is one of the most notable complications of pneumonia in pregnancy. The incidence of preterm labour reportedly has reached almost 44%, and, for preterm births, this figure is 36% [3]. Another recent population-based study revealed that the adjusted odds ratio for preterm birth in pregnant women with pneumonia was 1.71; moreover, the figures for low birth weight and small size for gestational age were 1.73 and 1.35, respectively [4]. Thus, pneumonia in pregnancy should be treated promptly and intensively using appropriate antibiotics.

*Mycoplasma pneumoniae* pneumonia is one of the common community-acquired respiratory tract infections and prevails among school-aged children and young adults [5]. Unfortunately, as far as we have searched, the exact incidence of* Mycoplasma pneumoniae* pneumonia in pregnant women is unknown. The major clinical manifestations are cough, fever, dyspnoea, and hypoxia. Generally,* Mycoplasma pneumoniae* pneumonia is treated using macrolides, and fulminant cases are relatively rare. However, the emergence and prevalence of macrolide-resistant strains have been a pressing challenge. The macrolide-resistance rates in adults are dramatically different from area to area: 3.5 to 13% in America, 0 to 10% in Europe, 3.3% in Oceania, 90 to 100% in Asia, and 56 to 89% in Japan [6]. The precise reason why this rate is very high in Asia, including Japan, is currently unknown. A plausible speculation is that macrolide-resistance* Mycoplasma* pneumonia was detected in Japan for the first time in the world [7], and the number of prescriptions of macrolide agents is high in China and Japan [8]. Importantly, macrolide-resistance strains are isolated more frequently from adolescent and paediatric patients than from adults; therefore, in adults, Japanese guiding principle still recommends macrolide for the treatment of* Mycoplasma pneumoniae* pneumonia. Meanwhile, a change of antibiotics to second-line drugs is also recommended if the fever does not subside in 48–72 h from macrolide administration [9].

In the case of macrolide-resistance in nonpregnant adult patients, tetracyclines or quinolones should be employed for the treatment of* Mycoplasma pneumoniae* pneumonia [9]. Because tetracycline exposure in utero causes congenital defects and permanent discoloration of bones and teeth, it should be avoided in pregnancy. Quinolones are a class of broad-spectrum antimicrobial agents that act by inhibiting bacterial DNA gyrase. Basically, definitive evidence for the teratogenic effect of quinolones has not thus far been shown. Instead, postnatal exposure to quinolones in juvenile mice and dogs caused arthropathy [10]. Thus, quinolones have been considered to be contraindicated, in principal, for pregnant women, although cartilage degeneration has not been reported in human neonate and children. In contrast, clinical data have been accumulated for decades that suggest the relative safety of quinolones in pregnancy.

The cohort study conducted by the European Network of Teratology Information Service reported that no clear adverse reactions, including birth defects, were revealed due to in utero exposure to quinolones [11]. Bar-Oz et al. conducted a meta-analysis and concluded that the use of quinolones during the first trimester of pregnancy did not appear to represent an increased risk of major malformations, preterm birth, or low birth weight [10]. Furthermore, according to the observational cohort study by Padberg et al., an increased risk of spontaneous abortion or major birth defects was not detected after intrauterine fluoroquinolone exposure [12].

Garenoxacin is a comparatively new oral quinolone that has a broad spectrum of antibacterial activities and shows the best antimycoplasmal activity among quinolones [13]. Additionally, according to the manufacturer's report, teratogenicity of Garenoxacin was not detected in rats or rabbits. Currently, the use of Garenoxacin in pregnancy is contraindicated despite insufficient clinical data. In the present case, however, the condition of the patient was deteriorating rapidly, and both maternal mortality and foetal mortality were concerned. Furthermore, the patient's gestational age, 13 weeks, was past the period of organogenesis. Consequently, after close consultation with the chest physician and patient, she fully understood the concerns about Garenoxacin usage in pregnancy and gave her written consent. Then, Garenoxacin was selected in expectation of definite effect.

In summary, we described a case of macrolide-refractory* Mycoplasma pneumoniae* pneumonia in pregnancy, in which administration of the oral quinolone, Garenoxacin, was highly effective. Although the use of quinolones has long been avoided in pregnancy, it can be justified with the emergence of drug-resistant bacterial pneumonia and in maternal life-threatening conditions.

## Figures and Tables

**Table 1:** The laboratory data of the case.

WBC	7,500	/*µ*L
Hb	12	g/dL
Ht	33.3	%
PLT	21.1 × 10^4^	/*µ*L
AST	14	IU/L
ALT	5	IU/L
LDH	170	IU/L
ALP	136	IU/L
TP	7	g/dL
Alb	3.2	g/dL
T-bil	1.2	mg/dL
Cre	0.43	mg/dL
BUN	7	mg/dL
CK	23	IU/L
Glu	88	mg/dL
AMY	43	IU/L
Na	133	mEq/L
K	3.4	mEq/L
Cl	98	mEq/L
CRP	8.8	mg/dL
